# HybridPLAY: A New Technology to Foster Outdoors Physical Activity, Verbal Communication and Teamwork

**DOI:** 10.3390/s16040586

**Published:** 2016-04-23

**Authors:** Diego José Díaz, Clara Boj, Cristina Portalés

**Affiliations:** 1 Industrial Systems Engineering and Design Department, Universitat Jaume I, Castelló de la Plana 12071, Spain; daz@uji.es; 2 Fine Arts Department, Universidad de Murcia, Murcia 30800, Spain; clara.boj@um.es; 3 Institute of Robotics and Information and Communication Technologies, Universitat de València, València 46010, Spain

**Keywords:** entertainment, physical activity, video games, children, playgrounds, ubiquitous

## Abstract

This paper presents HybridPLAY, a novel technology composed of a sensor and mobile-based video games that transforms urban playgrounds into game scenarios. With this technology we aim to stimulate physical activity and playful learning by creating an entertaining environment in which users can actively participate and collaborate. HybridPLAY is different from other existing technologies that enhance playgrounds, as it is not integrated in them but can be attached to the different elements of the playgrounds, making its use more ubiquitous (*i.e.*, not restricted to the playgrounds). HybridPLAY was born in 2007 as an artistic concept, and evolved after different phases of research and testing by almost 2000 users around the world (in workshops, artistic events, conferences, *etc.*). Here, we present the temporal evolution of HybridPLAY with the different versions of the sensors and the video games, and a detailed technical description of the sensors and the way interactions are produced. We also present the outcomes after the evaluation by users at different events and workshops. We believe that HybridPLAY has great potential to contribute to increased physical activity in kids, and also to improve the learning process and monitoring at school centres by letting users create the content of the apps, leading to new narratives and fostering creativity.

## 1. Introduction

Game-based technologies for entertainment and/or edutainment are not only for adults, but a great market exists centered on the early ages. A recent study [1] reveals that 38% of children under the age of 2 have used a mobile device for playing games, watching videos or other media-related purposes. By the age of 8, 72% of children have used a smartphone, tablet or similar device. By contrast, the use of playgrounds, ballparks, *etc.* decreases from the age of 6, which contributes to reduced physical activity and increased childhood overweight/obesity and creates a rift with children’s social environment.

From the social point of view, games are proven learning mechanisms—fundamental at early ages—to acquire skills and abilities such as communication and social interaction, and especially to be temporarily transformed into “other” people and acquire the ability to understand the view of the “other”, and thus substantially enhance our ability to understand the world and how to access it [2]. Although these benefits are evident in traditional games, in video games the problem is that their prolonged use can potentially lead to develop some addictions, especially in the so-called massive multiplayer online role-playing games, with the consequent risk of isolation and inability of children to develop social and communicative relations in the physical space; especially when children and adolescents, which are used to communicate through avatars in the digital networks, online games and virtual communities, have to do so in person [3,4,5,6,7]. Therefore, we understand that some video games can potentially generate a social and communicative isolation, everything unlike traditional games developed in public spaces such as catch, hide-and-seek, hopscotch, *etc.*, where children have to communicate verbally and physically with each other.

Different researchers and developers have dealt with one or some of these problems from different perspectives, e.g., by contributing to promote a healthier lifestyle with the development of technologies and media games that foster physical activity through the game, or by reducing the social break with the development of multiuser and collaborative games. In this sense, we can mention some game platforms such as Nintendo Wii and the games Wii Sport or Wii Balance Board, that revolutionized the gaming world by requiring users to physically exercise while playing or by converting them into personal trainers. Also, the Wii remote controller has been used by the research community to develop other applications and to investigate novel human-computer interactions [8,9,10,11]. A more recent example is Kinect, a range-based sensor from which many applications in the areas of entertainment and edutainment have been developed [12,13,14,15]. These platforms promote both physical activity and collaborative gaming, thus contributing positively to address the aforementioned problems. However, these platforms are still intended to be played indoors and require the use of other equipment such as a TV screen, and thus are not able to comprise the whole problem, as these facts restrict children’s playing freedom.

On the other hand, other research works and commercial solutions exist that merge playgrounds with virtual worlds, and thus promote physical activity outdoors. For instance, the interactive installation Water Games [16], is an excellent example of interactive outdoor gaming in which different dynamics are developed among large groups of users and where intensive multi-user interaction occurs. Another example is the Reactive Active Playground initiative [17]. In this research, new interaction and game systems were developed, which highlight the value that game systems designed with an open nature have for kids [18]. In such systems, a rich and dynamic partnership of children with the playing device occurs, because they personalize it with their own design of a game mechanism around the technological device and, at the same time, the game encourages their intellectual and creative development. Other similar examples are the Playware [19] and InnoPlay [20] projects. At the market level, the company Playnetic [21] offers a variety of products that allow interaction with different elements of the playground. To that end, they have created some elements with integrated technology that incorporate sound stimuli and that allow kids to, e.g., chat with each other, maintain songs playing while jumping in a stone, listen to stories after spinning a pendulum to generate energy in a storyball, *etc.* There exists other commercial solutions, such as Kompan Icon [22], i-play [23], Neos 360 [24,25] or Sona [26]. In all of them, the technology forms part of the elements of the playground. Finally, we would like to mention one of our previous projects called Zona de Recreo [27], first released in 2001, which consists of a multi user interactive platform arising from the transformation of a saucer swing that incorporates audiovisual digital technologies. The individual or collective use of this physical game interface, which we call video swing, allows the user to interact with a 3D virtual game by using the motion of his/her body. This application can be seen as one of the first solutions that incorporated technology (video games) in playgrounds.

Building upon the growing development of ubiquitous technologies and distributed computing, in our research we propose a paradigm shift by integrating the two media (virtual and physical) and generating smart, enhanced environments that engage children and youth to develop their physical and socio-communicative skills through a new game-based technology incorporated in the playgrounds. In this paper we present HybridPLAY, a novel technology that brings together the best of both worlds: firstly, it offers the full potential of the digital gaming industry (leisure linked to learning), and secondly it fosters outdoor physical activity (e.g., at playgrounds), without forgetting the collaborative (face-to-face) interaction. HybridPLAY is a new technology that enhances the urban space, opening new avenues for fun and gaming. Differently from the aforementioned solutions that merge playgrounds with virtual worlds, HybridPLAY is not integrated in the elements of the playground, but attached to them. In this way, kids can carry the HybridPLAY sensor and play with our proposed technology not only at every park, but also outside them (e.g., in the classroom, at home, *etc.*).

## 2. Materials and Methods

**Figure 1:**
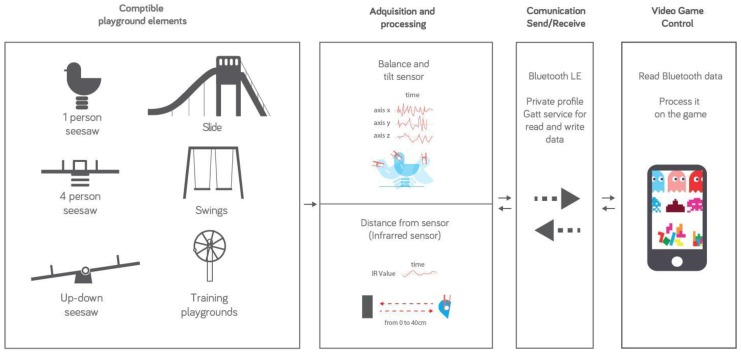
HybridPLAY workflow.

The HybridPLAY platform transforms playgrounds into interactive, game scenarios (Figure 1). This is achieved with a network of wireless sensors that, attached to playground elements, transforms them into physical interfaces that control video games (or apps) integrated in mobile devices. The sensors register the kids’ movements while playing on the swings, slides, see-saws, *etc.*, which are transformed into actions that control the digital game: walk, jump, run, turn, beat, *etc.* HybridPLAY combines physical and digital interaction to create outdoor gaming experiences by mixing digital game strategies with street game dynamics, verbal and corporal communication and team playing. Thus, with the HybridPLAY platform we aim at fostering outdoor physical activity and enhance communication and teamwork among players. Technically, HybridPLAY consists of a distributed network of sensors, easily adaptable to the gaming furniture of the playgrounds. These sensors wireless communicate with a server that handles the communication between them and the gaming terminals with which children can access the digital world. In this way, we have created a set of tools that transform playgrounds by means of a rapid, non-intrusive and reversible way.

While designing the sensorial elements of HybridPLAY, the following five key points have been taken into account:

1. Versatile placement: the sensors can be easily placed on any playground element without instructions, taking into account the diversity of materials, current condition, surface finishes, dimensions, and positions.
2. Intuitive single solution grip: HybridPLAY is mainly for kids, so it requires an intuitive grip, allowing children to easily change the game whenever they want with minimal installation time.
3. Customizable and suitable for all ages: though initially intended for children, HybridPLAY is versatile and has an immense range of potential users.
4. Compact and weather-resistant: naturality and impulsiveness are key in our games, so there’s no time for accessories.
5. Simple Design from a single mould: a simple design lets us keep costs down, and makes the project viable.

**Figure 2:**
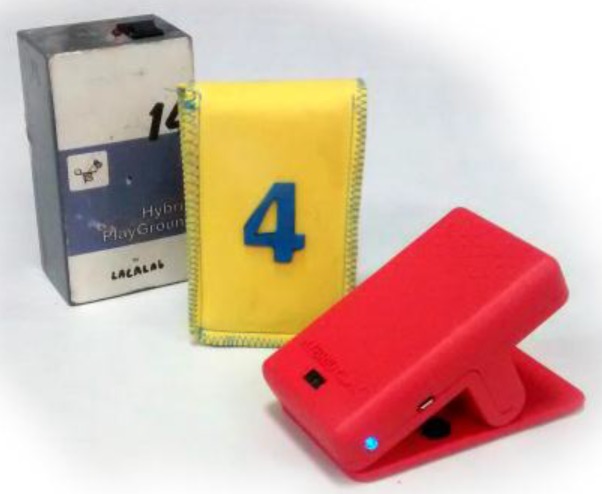
The HybridPLAY sensor. From left to right, the first, second and third versions.

Following these five points, since its first release in 2007, the sensor has undergone some improvements, leading to different versions (Figure 2). In the same way, the video games have also evolved, based on the valuable feedback of targeted users (see Section 4). A brief description of the different versions is shown in the following sub-sections.

### 2.1. First Version

**Figure 3:**
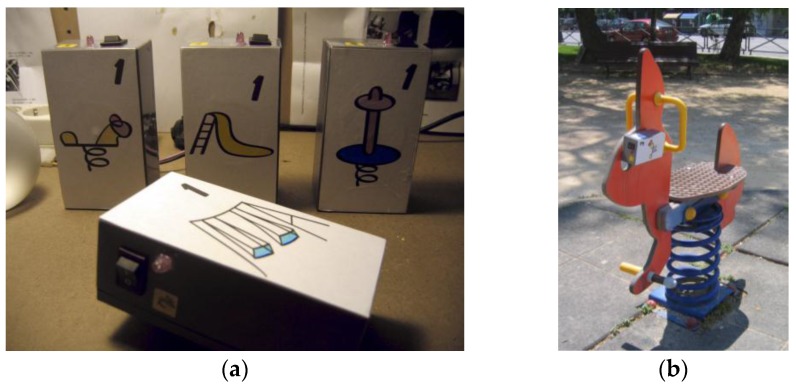
First version of the sensor. (**a**) The different models, according to the elements of the playground; (**b**) one of the sensors attached to the hobbyhorse.

The first version of the HybridPLAY sensor (released in 2007) basically consisted of an Arduino mini board [28], a three-axis accelerometer and an XBee module for the wireless communication [29]. In this first version, four different models of the sensor were produced according to different elements of the playground: the swing, the slide, the seesaw and the hobbyhorse. In all of them, the housing was composed of a plastic rectangular box (see Figure 2), which was afterwards covered with cardboard (Figure 3a) and stuck to the elements of the playground with an adhesive Velcro strip (Figure 3b). For the wireless connectivity we use the XBee protocol to create a mesh of sensors that send the information to a Linux laptop provided with an external XBee antenna. The laptop acts as a server with a custom-made Python server software that analyzes the info of the sensors, save the players’ scores and forwards the sensor triggers via WiFi to the player.

**Figure 4:**
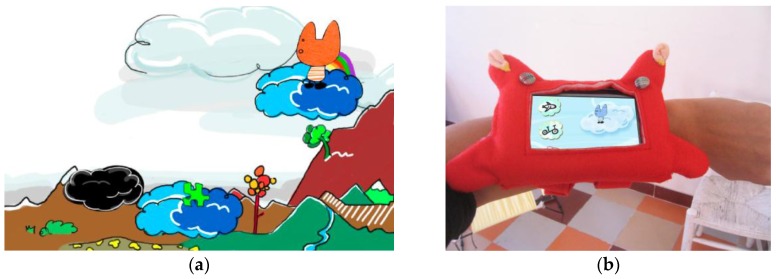
Video game example: (**a**) screenshot of the game Puzzle City; and (**b**) bracelet to hold a mobile device.

The first video games were designed by Clara Boj, and programmed by Diego Díaz and Martín Nadal with pygame [30]—a Python game library—and run on a Nokia N850 PDA with the Maemo Linux-based operating system, and produced by Intermediae Matadero Madrid (Madrid, Spain). The image in Figure 4a shows our first video game called Puzzle City, which consists of a set of mini games where the character has to collect the pieces of a puzzle to collect points and fill a gap created in the (virtual) city. When players fill this gap, they discover that the virtual world represents a playground. Each mini game is made up of simple dynamics associated with the movement of the child in the park, for example (Figure 4a) the character has to jump between (virtual) clouds by sliding down a (real) slide, where some clouds contain pieces of the puzzle and others none.

The mobile device running the video game was wearable, as it was integrated of a self-designed bracelet (Figure 4b). The system worked with four teams simultaneously, each one with a different bracelet color: blue, orange, red and green.

### 2.2. Second Version

**Figure 5:**
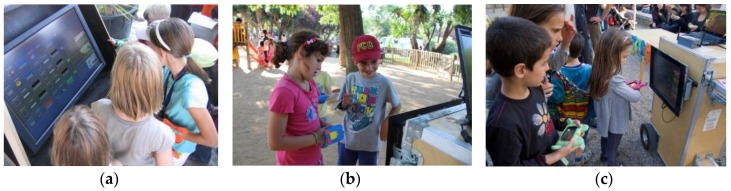
Kids looking the score in the server screen (**a**–**c**). Details of the bracelet with the integrated RFID tag (**b**).

The second version of the HybridPLAY sensor (released in 2009) was designed by Miguel de las Heras (Hangar, Barcelona, Spain), and was based on the technical specifications of the first version, to which some improvements were added. In particular, all the electronic elements were integrated into a single electronic board and some components were replaced. The main components of the HybridPLAY sensor in this version are: an ATmega168 processor (compatible with Arduino), a mini-USB connector, a three-axis accelerometer, an XBee component, a sound component and RFID reader and antenna. These improvements allowed us to reduce the dimensions of the sensors and the packing box, which also consisted of a rectangular plastic box that was stuck to the elements of the playground with Velcro. The video game (Puzzle City) was improved according of the feedback of kids. For example, we introduced the multiplayer option, where four teams can play the same game together and the laptop, that acts as a server, shows the score of each team, which is identified with a different color (blue, orange, red or green) according to the bracelet worn (Figure 5). Each team was composed of five members, one of them acting as coordinator, who carried the PDA on a bracelet in such a way that he/she could see the screen at all times to provide precise instructions to the other four team members. These members were running through the park by driving the physical elements in order to control the video games. As the implemented video game is made up of various mini games, the role of the coordinator was swapped among players for each mini game. We also designed new games based on kids’ ideas, improved the game speed and cleaned up some computational errors detected in the first version.

### 2.3. Third Version

**Figure 6:**
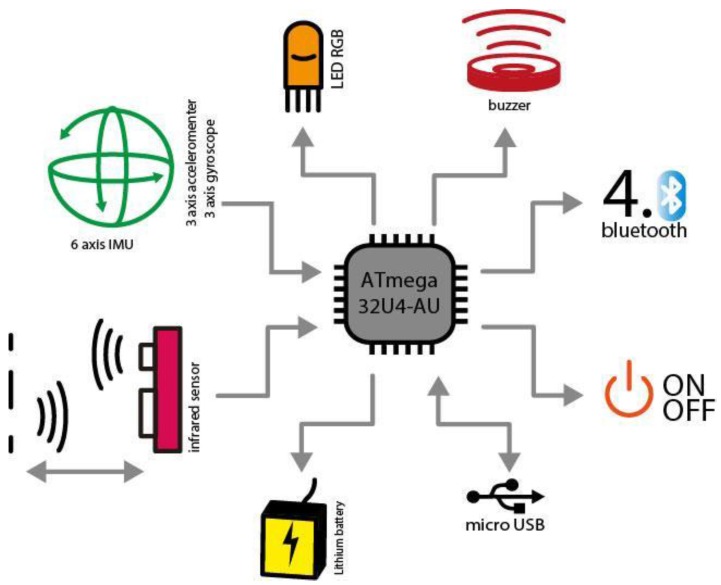
Technical components of the third version of HybridPLAY.

The current version of the HybridPLAY is the third version (released in 2015) [31]. In this case, the electronics of the sensor were also designed by Miguel de las Heras (Hangar, Barcelona, Spain), and consisted on the optimization of the components from the second version. The main components of the HybridPLAY sensor are, in this version (Figure 6): an ATmega32U4-AU processor (compatible with Arduino), a USB micro connector, a three-axis accelerometer and gyroscope, an infrared proximity sensor, a LED button and a sound component. In this case, the connectivity is achieved with Bluetooth LE (4.1).

**Figure 7:**
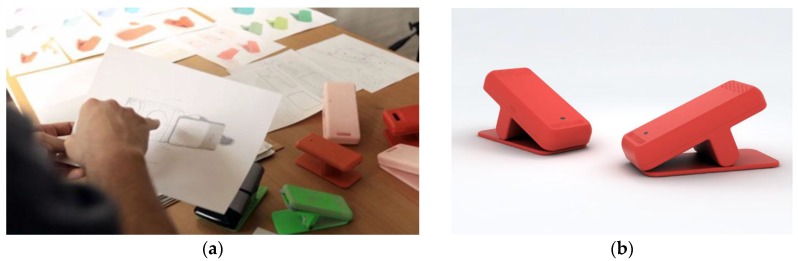
Image of the design process and different prototypes (**a**) and the final design (**b**).

The sensor case was designed by Joan Rojeski’s design studio with due consideration of the way children interacted with the different elements of the playground. Every decision was supported by prototypes adapted to the strength and dimensions of both children and adults. After several iterations and size adjustments of the clip system, a balanced solution was found, as shown in Figure 7.

**Figure 8:**
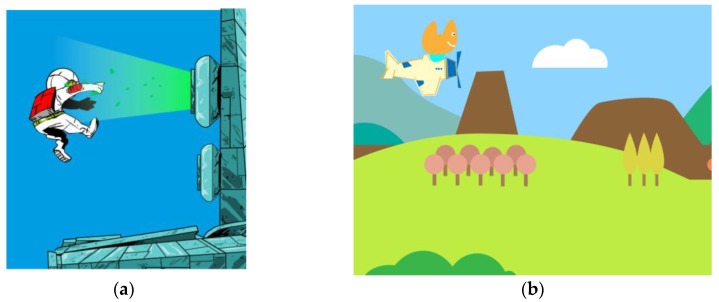
(**a**) Space Kids and (**b**) Puzzle City 2.

Regarding video games, our primary goal for this third version was both to improve the previously developed games (art, design, coding) as well as designing new games for the platform. The games for the third version were developed for both smartphone and tablet devices, and for both Android and iOS platforms, and initially we used the game engines cocos2dx and Unity. In this version the device and the sensors are connected directly via Bluetooth LE, so there is no further need to use the laptop server and the XBee protocol. The app was designed by Clara Boj and Diego Díaz, and programmed by Emanuel Mazza and Diego Díaz. This app includes different video games like Space Kids or Puzzle City 2. In Space Kids (Figure 8a), children can clean the galaxy by collecting space debris, explore a space station by balancing on the seesaw, or experience zero gravity by playing on the swings. In Puzzle City 2 (Figure 8b) children aim to find puzzle pieces to discover what is missing in the city by exploring the neighborhood with his/her team, walking around, and learning what different city spaces are meant for. The app also reinvents classic video games such as Pac-Man and Pong. Kids are aimed to play Pong on the swing, or help Pac-Man to escape moving their body on the seesaw.

The physical movements of the elements of the park, triggered by children, and their representation in video games, have a direct correlation to facilitate and optimize the dynamics of the game, with the aim that these dynamics based on movements become more intuitive, respecting and helping children to develop their spatial logic. 

For instance, when a child tilts the see-saw to the right, the character in the game moves to the right, and when he tilts it forward, the character moves up, *etc.* In the case of the slide, the character jumps when the child slides. In the case of swing, the character moves synchronized with its movements, drawing a circumference arc. Therefore, on the one hand, the actions that the child has to perform in the playground are the same or very similar to those that he would do to play without electrical devices and, on the other hand, the physical actions have a logical and intuitive correlation in the video game. In Table 1, a summary of the technical specifications for the three versions of the sensor is listed. As it can be seen, each version improves the capabilities of the former.

## 3. Interface Design

In the following sub-sections, the technical specifications of the interface design regarding Bluetooth communication, frame rate, sensoring and user interaction are described in detail for the current version of the system—the third version. Within this, we aim at providing detailed information of the input/output parameters of the sensors and how the interaction is produced according to the different elements of the park.

### 3.1. Bluetooth Communication

In order to communicate the sensor with the mobile devices, the Bluetooth Low Energy (BLE) or Bluetooth Smart protocols are used, both belonging to Bluetooth 4.0. The chip used is the RN4020, which is internally programmed to accommodate a private GATT service with three characteristics: NOTIFY (11 bytes), WRITE (2 bytes) and WRITE (2 bytes). In the characteristic NOTIFY, a string is recorded in hexadecimal form, which contains the following values from the sensors: IR (1 byte), BATTERY (1 byte), TILT (1 byte) and QUATERNIONS (8 bytes), where IR stands for the infrared proximity sensor. All of these values are normalized in integer values. In the case of IR and BATTERY, the values go from 0 to 100; TILT is a Boolean, so it can be 0 or 1; and QUATERNIONS go from 0 to 255.

The primary difference with a standard communication through the serial port (used for example in the classic Bluetooth 2.0) is that, in the case of using private services and features, there is already a “container” prepared for the variable, which is directly accessible. By contrast, with a standard reading of the serial port, the data must be packaged and temporized to avoid overflow. For instance, it is common in a standard script to add a header to the message and to add a control on the reception in order to start reading only after receiving the header; this can lead to eventual jumps in reading because, if mistakenly the header is not automatically read, the rest of the message is lost until the next refresh. With the private services of the BLE, this problem disappears.

### 3.2. Frame Rate

Regarding the electronics, the firmware internal clock is set to 25 Hz, which corresponds to 25 refreshes per second or a single refresh every 40 ms. The refresh rate is the same for all sensors. Regarding the application, a new reading of the values recorded in the feature NOTIFY (which records all sensor data) is performed at each update cycle.

### 3.3. Sensoring

To retrieve the rotations of the sensor, the quaternion system was used, which is represented by the parameters *q*_0_, *q*_1_, *q*_2_, *q*_3_. Though less intuitive than Euler angles, quaternions provide an alternative measurement technique that does not suffer from gimbal lock, which arise when two of the three axes are aligned and a degree of freedom is lost. The relationship between quaternions and the Euler angles ϕ (roll), θ (pitch) and ψ (yaw) can be defined as follows:

(1) $$\left[\begin{array}{c} \phi \\ \theta \\ \psi \end{array}\right]=\left[\begin{array}{c} arctan\frac{2\left(q_{0}q_{1}+q_{2}q_{3}\right)}{1-2\left(q_{1}^{2}+q_{2}^{2}\right)}\\ arcsin\left(2\left(q_{0}q_{2}-q_{3}q_{1}\right)\right)\\ arctan\frac{2\left(q_{0}q_{3}+q_{1}q_{2}\right)}{1-2\left(q_{2}^{2}+q_{3}^{2}\right)} \end{array}\right]$$

where the resulting angular values are between −180° and +180 for ϕ and ψ, and between −90 and +90 for θ.

The TILT parameter is internally detected by the firmware after a calibration (some consecutive lectures of the accelerometer) when the sensor is initialized. This calibration value is continuously compared with the data of the gyroscope and the accelerometer to detect if they go outside a defined range, in which case a sudden movement is detected. The accuracy of the TILT depends both on the sensor and on the defined tolerance level. The infrared proximity sensor that is currently being used, the GP2Y0E02B, has a detection range between 3 and 100 cm. Beyond this range, the sensor is set to inactive.

### 3.4. User Interaction

#### 3.4.1. Springy Elements

**Figure 9:**
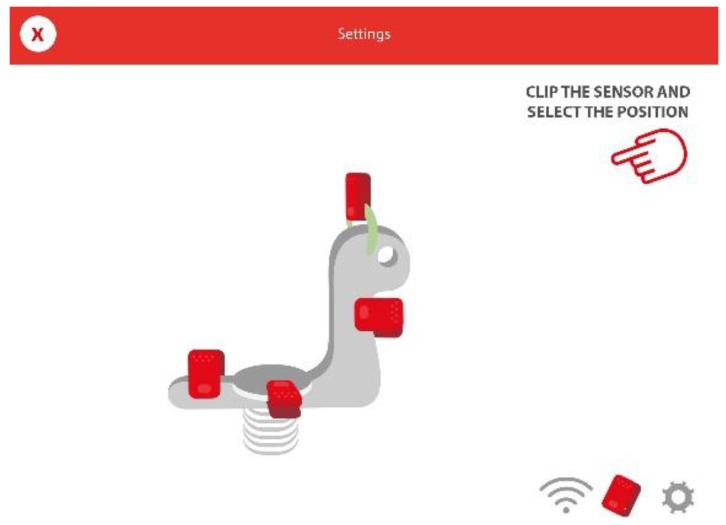
Instructions to perform the calibration of the sensor with regards to the element of the playground.

When attaching the sensor on any element of the playground that is provided with one or more springs (e.g., seesaw or hobbyhorse), the behavior of the sensor is similar to the traditional joysticks. Initially, a simple but effective calibration process is carried out (Figure 9). First, the sensor is coupled in any of the orientations indicated in the app, and the user selects in the app the chosen position.

Once this configuration is finished, when starting the game, the system automatically calibrates the sensor with respect to the inclination it has. For a proper calibration, it is necessary that the element of the park is as still as possible.

**Figure 10:**
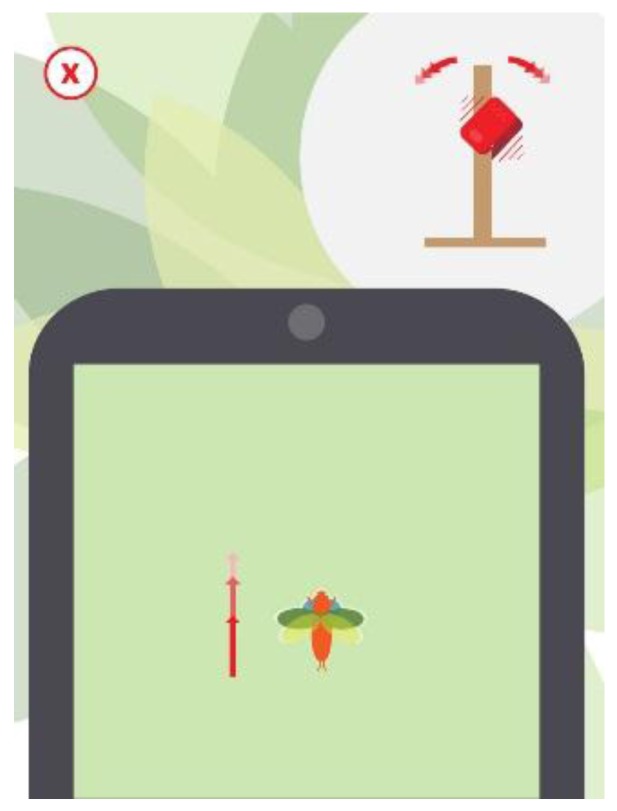
Screenshot of the video game Moskis.

Thanks to its sensor, two general types of motion can be detected: tilt and inclination. With the tilt, sudden movements are detected, and the seesaw/hobbyhorse game dynamics associated with it are established, such as increasing energy or the value of a variable of the player. For example, in the case of Moskis, the fly begins its flight when tilting the element of the park (Figure 10).

**Figure 11:**
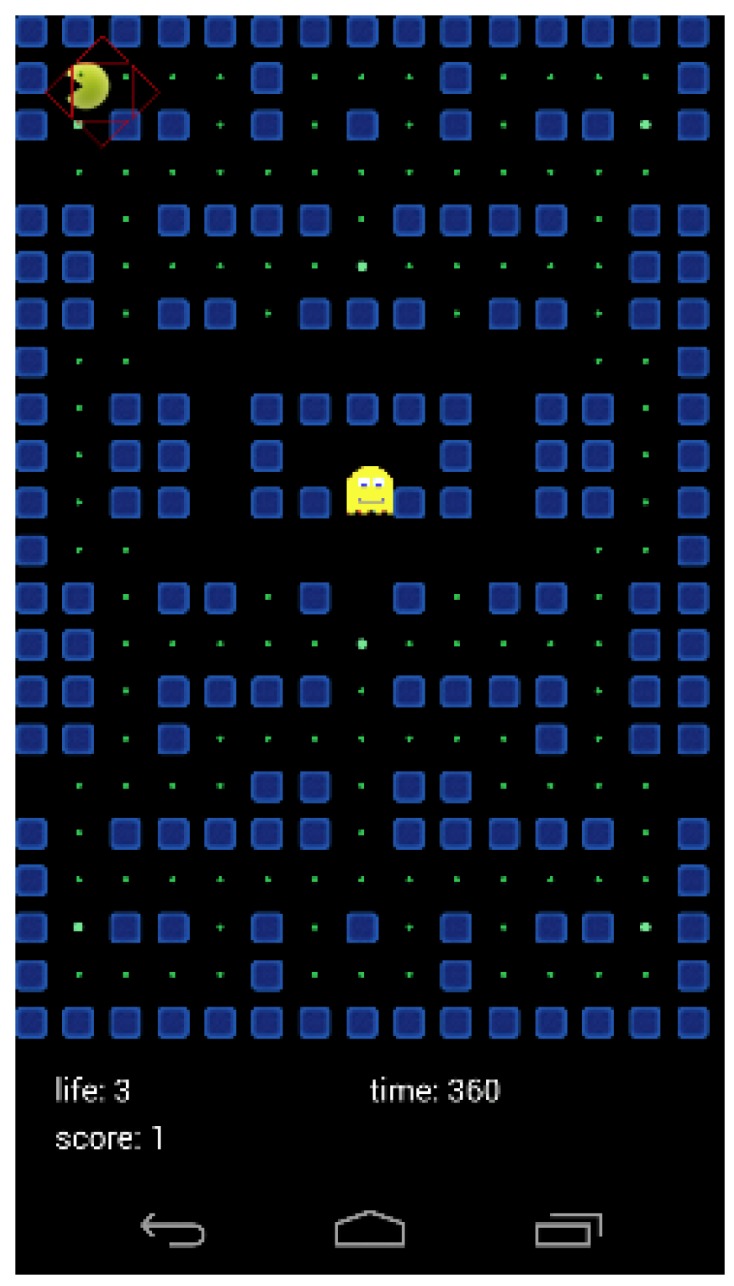
Screenshot of the adaptation of the classic arcade video game Pac Man.

The 4-way movement is more complex, and is detected when there is a significant variation in ϕ (tilt left–right) and θ (tilt forward–back) with respect to the values recorded in the initial calibration. These movements are used in video games to control the direction of the user, often on a two-dimensional plane in an aerial view, such as in the mazes of the adaption of the classic arcade video game Pac Man (Figure 11).

The effect of the inclination in the game can be digital (activating a predefined movement speed) or analog, so that the speed of the associated action is related to the value of the inclination angle.

#### 3.4.2. The Swing

**Figure 12:**
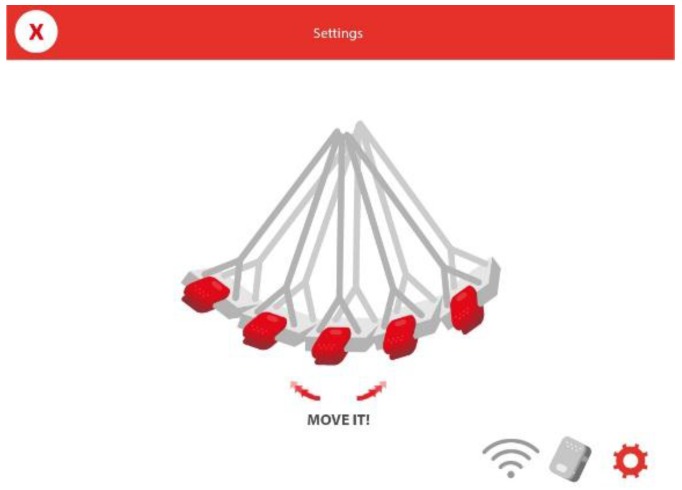
Instructions on how to trigger interaction with the swing.

In the case of the swing, the sensor must be coupled to the base thereof facing the floor, so that it can be detected. When the child sways, the sensor recognizes everytime that the swing is closer to the ground (Figure 12). In this way, the swinging speed can be calculated, since the cutting period is higher or lower depending on it.

**Figure 13:**
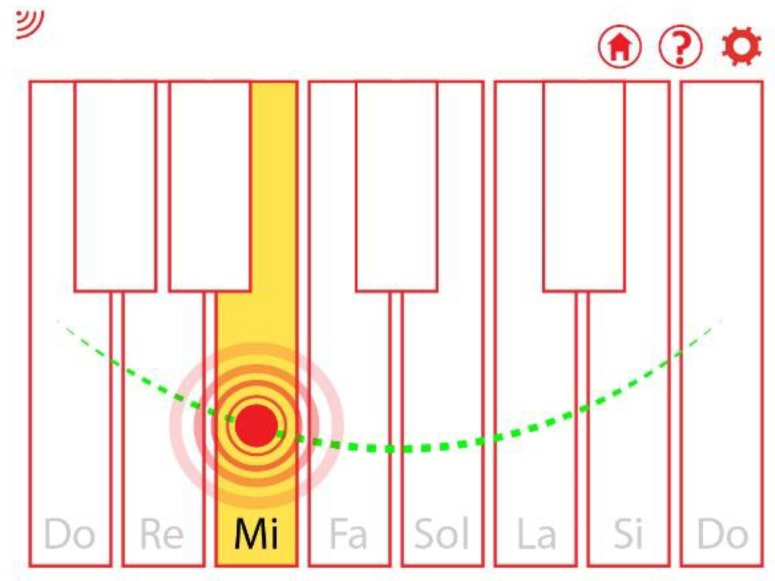
Example of the interaction produced in the game HybridEDU with the swing.

These movements can be reflected in the actions of the app in different ways, for example to control the speed of the protagonist or to also control its position, moving from left to right of the screen, in a linear motion, or forming an arc. An example is depicted in Figure 13, where the movements of the swing produce an interaction in the video game HybridEDU (in this example, intended to play a piano).

#### 3.4.3. The Slide

**Figure 14:**
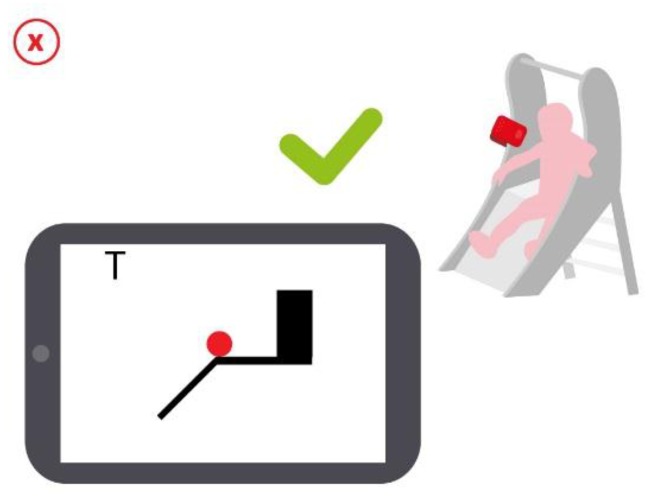
Schematic of the interaction produced by placing the sensor on the slide. The system detects that the child is waiting for jumping.

In this case the sensor must be attached to the side of the slide, so that the infrared proximity sensor is facing the child, sensing his presence when sitting at the top of the slide. To ensure that the child is waiting to pounce, the app detects whether the infrared proximity sensor has been cut for at least 2 s. Once the presence of the child has been detected, a signal appears in the game indicating that the system is ready to detect his jumping (Figure 14).

Regarding to the child jumping to the slide, it is only detected the instant when it occurs, but not the speed or time sliding down the slide. Other actions may be associated with the activation of any event, such as a shot or the game's protagonist jump.

#### 3.4.4. Other Possibilities of User Interaction

The games designed for HybridPLAY can also use the classical mechanisms of interaction provided on smartphones, such as the interaction with the screen through the touch (single touch or multi touch) or the internal accelerometers. The actions associated with these interactions are the same as in other video games for mobile devices, but are of particular interest and relevance as they complement those of the HybridPLAY device, as the child with the role of coordinator has to order the actions to be taken, at the same time that he/she interacts with the game.

## 4. Evaluation and Discussion

Since its beginnings in 2007, the HybridPLAY system has been tested and exhibit in many different places, from artistic exhibitions to conferences and workshops. Altogether, the HybridPLAY system has been tested by around 1500 children and 400 parents (estimated values). Based on the feedback of these trials, the sensor has evolved from its first to the current version. The complete list where HybridPLAY has been tested/exhibited up to date is provided in the following points, according to its different versions. The approximate number of people (children and adults) that tested the system is also given. The first version was exhibited and/or tested in:

- Utopias, artistic residence, Intermediae Matadero (Madrid, Spain, 2007)—30 children, 10 adults
- A new economy, Ars Electrónica Festival (Linz, Austria, 2008)—40 children, 20 adults
- Amber 08 Festival (Istanbul, Turkey, 2008)—50 children, 10 adults
- Hybrid Playground, Techformance Festival (Murcia, Spain, 2009)—100 children, 30 adults
- Techformance, Arco 09, Stand Región de Murcia, (Murcia, Spain, 2009). Exhibition
- Hybrid Playground, LAboral Centro de Arte y Creación Industrial, (Gijón, Spain, 2009)—300 children, 60 adults

The second version was exhibited and/or tested in:

- Hybrid Playground, Mobilefest 09 (Sao Paulo, Brazil, 2009)—200 children, 50 adults
- Taller Hybrid Playground (Valdemoro, Spain, 2009)—60 children, 20 adults
- Hybrid Playground WIP 09, (Barcelona, Spain, 2009)—40 children, 0 adults
- Hybrid Playground, MuPAI, Museo Pedagógico de Arte Infantil (Madrid, Spain, 2010)—0 children, 40 adults
- Hybrid Playground, PAM, Cal Masó, (Tarragona, Spain, 2010)—40 children, 10 adults
- Hybrid Playground Hó Play 2010, Certamen internacional de videojuegos independientes, (Bilbao, Sain, 2010)—40 children, 10 adults
- Paisatge?, Bolit La Rambla, (Girona, Spain, 2010)—200 children, 60 adults
- Create your world, Ars Electrónica Festival 11 (Linz, Austria, 2011)—50 children, 20 adults
- Mercé Arts al Carrer (Barcelona, Spain, 2012)—200 children, 100 adults
- Games on public space: Hybrid Play, Conference at School of Software and Microelectronics, Beijin University, (Beijin, China, 2014).

To date, the third version has been exhibited in:

- Workshop in TEDx Barcelona education (Barcelona, Spain, 2015)—0 children, 30 adults
- Workshop Medialab-Prado (Madrid, Spain, 2015)—12 children, 25 adults
- Workshop La Fira d’Oci Infantil i Juvenil de Nadal, (Castellón, Spain, 2016)—100 children, 50 adults

### 4.1. Workshops

The performed workshops with group of children have had an approximate duration of two hours each, and have been organized as indicated in Table 2.

The experience of the workshops was very positive. From the first moment, children were quite engaged with the proposed games, and enjoyed the system very much. However, despite the great success of the system in the first version, we found some aspects that needed to be improved, which were based on our personal observations and the feedback from parents and children.

Regarding to the participation of children, we noticed that children between 6 and 10 years old responded better to the system, while children under 6 didn’t have enough strength to move the spring elements and also experienced some difficulty in understanding the dynamics of the games children over 10 tended to feel very attracted to the system, but the available games seemed too simple to them. Besides, some children under 8 had difficulty discerning between left and right, and also in being to swing on the swings without the help of another child or adult. Regarding the task of coordinator, children performed differently according to their personal profiles: while some of them felt more comfortable doing this task, others (more introverted) had a harder time at first, but soon developed well in this task. We also found no integration problems with the other children of the park that were not playing with HybridPLAY, so we can say that the system does not interfere with the normal use of the park by other children. Additionally, after finishing the workshops, many children continued playing in the park imagining they were doing that with HybridPLAY, and also brought their own ideas for games to take place, and thus we can conclude that the system encourages imagination and creativity. Also, parents were easily engaged into the dynamics of the game, and so HybridPLAY functions as an integrator of family activity at playgrounds.

Besides these good results, there were other issues and technical aspects that could be improved. For instance, regarding the game dynamics, it would be interesting to compete between teams for a global gaming session in order to increase user engagement and game capabilities. Regarding the technology itself, we found that the technological system used in this first version was not very portable and lacked complete autonomy (e.g., short battery life). Regarding the proposed video games, it would be interesting to add other elements, such as animations. We were also conscious that the videogames responses were slightly slow (mainly due to the mobile technology) and that some bugs had to be corrected.

All the aforementioned negative aspects were tackled in the second version of HybridPLAY. The autonomy and portability of the system was greatly improved. In this regards, we constructed a portable cart to transport all the equipment, which was equipped with a car battery and an inverter to provide power to the laptop and recharge the batteries of the sensors and PDAs. When the car was unfolded, a 24-inch flat screen showed the players the status of their game, the mini game they were playing and the points scored by each of the four teams. This display was very well received as children continually ran to the car to see the status of the game.

The workshops carried out with the second version of the system also had great acceptance by children and parents. Additionally, we found that the game dynamics were more fluent due to the improvements that were made. For instance, the improvement of the battery life and the absence of inconsistence errors in the video games was deterministic. In this way, the game was not interrupted at any time, leading to a greater user engagement. Also, the addition of animations in the video games and the possibility to compete among teams was greatly accepted by both children and parents, as the gaming possibilities augmented, as well as the teamwork. However, other issues were found which motivated other modifications and improvements, leading to the third version of the system.

In the third version, we included more complete and sophisticated dynamics in the videogames, as well as sound output and more attractive animations, also increasing the speed response. Regarding to the technology, the autonomy and portability of the system was further increased (allowing 20 h of continuous play), while the server computer was eliminated, and hence also the need to use the car with the car battery and the inverter.

The improvements made in the third version lead to a pre-industrial system, with all the aforementioned encountered issues addressed. The feedback after the last workshop carried out to date (TEDx Barcelona) make us think that HybridPLAY has great potential in the entertainment, education and fitness markets, and that the targeted users are not restricted to children, but also e.g., parents are engaged in the game.

### 4.2. Exhibitions

**Figure 15:**
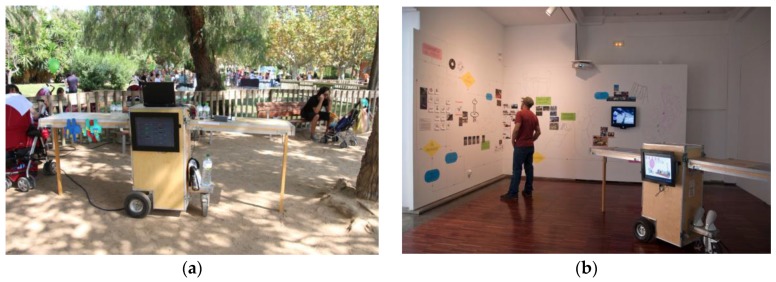
Cart of HybridPLAY in the park (**a**) with the monitor showing the score of the games and at one of the exhibitions (**b**) with the monitor showing a loop of the video games.

The HybridPLAY system was presented at some artistic exhibitions, attracting the curiosity of the general public. In Figure 15b the system is depicted at the Techformance Festival (Murcia, Spain, 2009). As this was version 2, the cart was used, which was designed with the purpose of augmenting the autonomy of the overall system. The cart was provided with a car battery and an inverter, that allowed us to increase the autonomy of the system from 2 to 6 h and also to recharge the PDAs. This was relevant for the exhibitions, where the system was on display for the whole day.

The cart had a set of drawers covered with foam to absorb impacts and to transport tools, the Velcro, sensors, batteries, inverter, *etc.* It was equipped with two doors that could be extended, forming a table and, when closed, it was compact enough and highly resistant to transport all the material, so it could be easily transported to different exhibition venues.

### 4.3. Quantitative Evaluation

A quantitative user evaluation of the HybridPLAY system was carried out during the Workshop La Fira d’Oci Infantil i Juvenil de Nadal, (Castellón, Spain, 2016) with the third version of the system. Around 150 people tested the system at the workshop, from which a total of 87 acceded to fill out a pair of questionnaires, that were related to the usability of the system and the individual’s satisfaction with the proposed activity, where the System Usability Scale (SUS) [32] was chosen to measure usability.

The participants were both adults (32 participants) and children (55 participants). The adults that participated in the survey were the parents of children, who often took the role of coordinators. In Table 3, the socio-demographic data of these two groups is provided, showing the mean, standard deviation (s.d.) and minimum and maximum age of the participants. Other parameters used are gender and how often they use video games, where the range 0–4 for the frequency (f.) means: 0: never or almost never; 4: every day. Most of the children participating in the questionnaires fulfilled them with the help of their parents and/or other adults supervising the activity. As we have detected slightly different results in the questionnaires regarding adults and children, so we present the results divided into these two groups. We have, however, not detected relevant differences regarding gender and video game use frequency.

The results of the SUS questionnaire are listed in Table 4, where the obtained scores are provided for the two considered groups, adults and children. In questions 1 to 10 the range 0–4 means: 0: strongly disagree, 4: strongly agree. The values of the SUS score, however, range from 0 to 100, meaning 100 the best imaginable result. For the adults group, this score reaches 84.92 points; for the children group, the score is slightly better, reaching 91.23 points. These values can be considered good (for adults) and excellent (for children) on the scale of scores provided by the questionnaire and taking into account the fact a minimum score of 68 would be deemed acceptable for a tool [33,34].

The results of the individuals’ satisfaction questionnaires are given in Table 5 for the two considered groups. The scores also range from 0 to 4, meaning: 0: strongly disagree, 4: strongly agree. As it can be seen, results are very satisfactory. As a general observation, it can be seen that the adults is more critical, as slightly worse scores are obtained for almost all the questions of the two questionnaires. Besides, the obtained results are excellent or almost excellent, what we believe is the consequence of the different improvements carried out for the HybridPLAY system, after listening to users’ feedback during the many organized workshops and exhibitions during the last years.

### 4.4. Extended Uses of HybridPLAY

**Figure 16:**
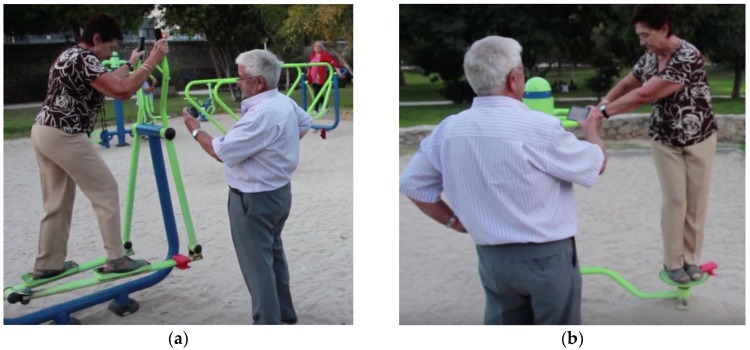
Seniors playing with HybridPLAY at the elliptical machine (**a**) and at the ab twister (**b**).

Although our main targeted audience are children, the HybridPLAY sensor can be also used by other users such as seniors. In Figure 16, an example is shown with the sensors placed at other elements of the playground that are intended to do exercise. In this case, the video games were adapted, in such a way that we simplified the graphics and game dynamics by using basic shapes and colors. We would like to stress the potential value of HybridPLAY to help the physical and verbal synchronization of seniors by motivating their mental activation and improving their response time to external stimuli.

**Figure 17:**
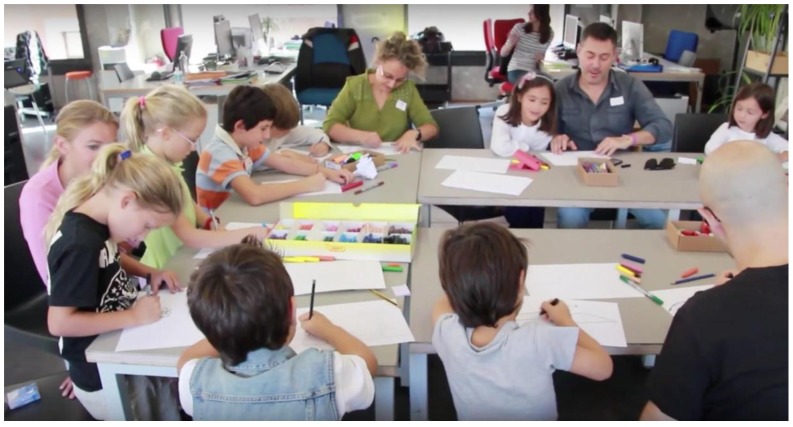
Children and parents designing their own games for HybridPLAY at the Medialab-Prado (Madrid, Spain) workshop.

The versatile character of HybridPLAY means that it is not restricted to be outdoor use, and indoor activities can also be performed. In this regards, HybridPLAY has also been tested at the classroom level (Figure 17) and extra school activities, where kids can enjoy and learn at the same time they are playing with HybridPLAY. We have developed a set of basic games with educational contents such as maths, where kids can learn basic addition and multiplication operations. So far, we have received a very positive feedback from this experience, maybe due to the fact that the physical activity helps the mental activation and the subsequent learning process.

The video games can be also customized, in such a way that the educational sector can benefit from HybridPLAY, including the education of kids with special needs. In this regards we have developed some basic experiences with kids with diminished mental capabilities and we have realized that in some cases, after properly adjusting the level of the game, our system can be very positive for their mental development.

## 5. Conclusions and Further Work

In this paper we have presented HybridPLAY, a new platform composed of sensor units and video games that fosters outdoor physical activity, verbal communication and teamwork. We have described the work performed to date, both at the technological and at the evaluation (user-related issues) level, that has led us to achieve a robust version of both the sensor and the video games. The results of a quantitative evaluation of the system carried out for its third version are excellent, what we believe is a consequence of the different improvements carried out for the HybridPLAY system, after responding to users’ feedback during the many organized workshops and exhibitions attended during the last years.

We also have demonstrated the versatile character of HybridPLAY. Though initially intended to foster outdoor physical activity in playgrounds, where the main targeted users are children, HybridPLAY can also be used by other audiences (e.g., seniors) and can be used indoors. Additionally, the video games can be customized in order to provide benefits in the educational sector, including the education of people with special needs.

HybridPLAY is a platform that started as an artistic initiative to address actual problems, that however has today reached great acceptance by the public and has become the primary product of Lalalab Projects. With a view the mass production and commercialization of the HybridPLAY platform, some steps are still required. In particular, regarding the hardware, we intend to reduce the dimension of the sensor; regarding to the software, we intend to build a platform that allow users to design their own games. We also intend to introduce the sensor in the entertainment, education and fitness markets, possibly customizing some of the features of HybridPLAY (e.g., video games) for the different sectors. Additionally, we believe that HybridPLAY can be used as a complementary tool to fight overweight and obesity in children, as it fosters physical activity. In order to demonstrate this claim, we plan to provide a group of children with our technology for two or three months, to track their physical activity and to evaluate the use they make of it.

## Figures and Tables

**Table 1:** Technical specifications and other characteristics for the different versions of the system.

	First Version	Second Version	Third Version
**Voltage**	5 V	5 V	3.3 V
**Batteries**	6 AA 25,000 mA 1.2 V (x9 batteries)	2000 mAh polymer lithium rechargeable battery	1000 mAh polymer lithium rechargeable battery
**Accelerometer**	3 axis ± 2 g	3 axis ± 3g	3 axis ± 16 g
**Gyroscope**	**-**	-	±2000 °/sec (dps)
**Infrared proximity sensor**	10–80 cm	4–40 cm	3–40 cm
**RFID**	**-**	125 kHz read frequency EM4001 64-bit RFID tag compatible	(optional) 13.56 MHz read frequency. MFC and PCD mode for ISO/IEC14443-3 Type B and ISO/IEC14443-4 Type B cards
**Wireless protocol**	XBee/WiFi	XBee/WiFi	Bluetooth 4.1 LE
**Full duplex communication**	no	no	yes
**Frame rate**	10 fps	15 fps	30 fps
**Delay (approx.)**	2000 ms	1500 ms	100 ms
**Game programming language**	pygame	pygame	cocos2dx/Unity
**Requires server**	yes	yes	no
**Supported handheld devices**	Nokia N850	Nokia N850	Android and IOs SmartPhone
**Waterproof**	no	no	IP55
**Impact resistant**	no	no	yes
**Adaptable to all elements in the playground**	no (four different designs)	yes	yes
**Ergonomic case design**	no	no	yes
**Sound**	no	yes (only the sensor)	yes (sensor and videogames)
**Complexity of animations**	low	medium	high
**Video games**	Puzzle City	Puzzle City (improved)	Space Kids, Puzzle City 2, Pac-Man, Pong, Moskis, HybridEDU

**Table 2:** Schedule for the workshops.

Duration	Description
-	Beginning. The group of children arrives to the playground
10 min	Children freely play with the elements of the playground. Observation.
5 min	Brief talk relating to the games in the playground. Some questions arise, such as: Do you usually play in the playground? What are your favourite elements of the playground? What element do you like the least? Is there any missing element which you would like to play with? Which one? Do you find funny the see-saw, the swing, *etc.*?
5 min	Brief talk relating to the workshop, explaining: The games proposed in HybridPLAY The role of children as avatars The responsibility of children regarding each other and the devices that they carry during the game The organization into two teams
10 min	The elements of the game: the way the HybridPLAY sensors are integrated in the different elements of the playground and how do they work.
5 min	Creation of gaming teams: Four groups of 3 to 4 players Two groups of observers These groups rotate, in such a way that every children becomes a player and an observer. Each group of players has a game coordinador, which is the child that holds the smartphone. For every mini game, the game responsibility changes, so all the kids will hold the smartphone and coordinate the team. The sensors are handed to the children.
20 min	Game experience: Children freely play with the HybridPLAY platform integrated in the elements of the playground. Observation. The observers do not participate in the game, but only see the actions of their colleagues.
5 min	Group rotation. Players become observers and *vice versa*.
20 min	Game experience.
15 min	Brief talk. Feedback from children. Some questions arise: What do you think about the game experience? Do you find it funny? Would you like to play more complex games following this system? Was it easy to understand how it works? As an observer, what have you seen? What kind of games would you like to play at the playground? What others improvements do you suggest for the system?
-	End of the workshop

**Table 3:** Socio-demographic data of the participants.

	Adults					Children				
Age	**mean**	**s.d.**	**min**	**max**		**mean**	**s.d.**	**min**	**max**	
	35.25	7.57	22	47		8.56	2.05	6	12	
Gender	**male**	**female**				**male**	**female**			
	14	18				31	24			
Videogame frequency (%)	**f. 0**	**f. 1**	**f. 2**	**f. 3**	**f. 4**	**f. 0**	**f. 1**	**f. 2**	**f. 3**	**f. 4**
	12.50	40.63	31.25	9.38	6.25	1.82	9.09	25.45	30.91	32.73

**Table 4:** Results of SUS questionnaire (mean, standard deviation, minimum and maximum).

	Adults				Children			
Questions	mean	s.d.	min	max	mean	s.d.	min	max
1. I think that I would like to use this system frequently	3.28	0.57	2	4	3.78	0.45	2	4
2. I found the system unnecessarily complex	1.19	0.73	0	2	0.55	0.71	0	2
3. I thought the system was easy to use	3.59	0.55	2	4	3.78	0.49	2	4
4. I think that I would need the support of a technical person to be able to use this system	0.41	0.55	0	2	0.38	0.59	0	2
5. I found the various functions in this system were well integrated	3.22	0.48	2	4	3.82	0.39	3	4
6. I thought there was too much inconsistency in this system	0.69	0.81	0	2	0.55	0.60	0	2
7. I would imagine that the most people would learn to use this system very quickly	3.72	0.80	1	4	3.75	0.44	3	4
8. I found the system very cumbersome to use	0.69	0.58	0	2	0.49	0.68	0	2
9. I felt very confident using the system	3.38	0.60	2	4	3.65	0.55	2	4
10. I needed to learn a lot of things before I could get going with this system	0.25	0.43	0	1	0.33	0.47	0	1
**SUS score**	84.92	11.48	52.50	100.00	91.23	11.05	65.00	100.00

**Table 5:** Results of the individuals’ satisfaction questionnaires (mean, standard deviation, minimum and maximum).

	Adults				Children			
Questions	mean	s.d.	min	max	mean	s.d.	min	max
1. I liked very much playing with the video games	3.69	0.46	3	4	3.84	0.37	3	4
2. I find the video games appropriated for my age and/or my children’s age	3.44	0.66	2	4	3.82	0.39	3	4
3. I liked very much the concept of playing with the elements of the park inside HybridPLAY	3.69	0.46	3	4	3.87	0.33	3	4
4. I find it very easy to collaborate with other friends in HybridPLAY	3.63	0.48	3	4	3.76	0.42	3	4
5. I would like to play more with HybridPLAY	3.72	0.45	3	4	3.87	0.33	3	4
6. I would like to recommend others to play with HybridPLAY	3.66	0.54	2	4	3.85	0.35	3	4
